# Assessment of disease activity in patients with rheumatoid arthritis using plasma tumour M2-pyruvate kinase test

**DOI:** 10.3389/fimmu.2022.901555

**Published:** 2022-08-18

**Authors:** Sung Soo Ahn, Hye Min Kim, Younhee Park

**Affiliations:** ^1^ Division of Rheumatology, Department of Internal Medicine, Yongin Severance Hospital, Yonsei University College of Medicine, Yongin, South Korea; ^2^ Department of Pathology, Yongin Severance Hospital, Yonsei University College of Medicine, Yongin, South Korea; ^3^ Department of Laboratory Medicine, Severance Hospital, Yonsei University College of Medicine, Seoul, South Korea

**Keywords:** tumour M2-pyruvate kinase, rheumatoid arthritis, disease activity, predictor, biomarker

## Abstract

**Background:**

Pyruvate kinase M2 (PKM2) is an enzyme that regulates the final process of glycolysis and exists in tetrameric and dimeric forms. The dimeric form of PKM2, also known as tumour M2-PK, increases when aerobic glycolysis is augmented, a feature observed in rheumatoid arthritis (RA). We investigated whether plasma tumour M2-PK is elevated in patients with RA and whether its levels correlate with disease activity.

**Methods:**

Plasma levels of tumour M2-PK were measured for patients with RA (n=151), those with osteoarthritis (OA) (n=37), and controls (n=37). We evaluated the association between plasma tumour M2-PK and continuous variables using Pearson’s correlation analysis, and multivariate logistic regression analysis to determine the association between plasma tumour M2-PK and disease activity status. Knee synovial tissue blocks from patients with RA and OA were subjected to real-time quantitative PCR (qPCR) using two different primers for PKM2 and tumour M2-PK immunohistochemical (IHC) staining.

**Results:**

The tumour M2-PK level significantly correlated with the disease activity score in 28 joints (DAS28)-erythrocyte sedimentation rate (ESR) (r=0.546, p<0.001) and DAS28-C-reactive protein (CRP) (r=0.589, p<0.001). Moreover, repeat testing of tumour M2-PK levels in 20 patients revealed a significant decline in tumour M2-PK levels after reduction in inflammation (p<0.001). Area under the receiver operating characteristic curve (AUROC) analysis demonstrated that upon incorporation of tumour M2-PK, ESR, and CRP, the area under the curve was 0.962 for distinguishing moderate/high from remission/low disease activity. Adjusted logistic regression also revealed that a tumour M2-PK >43.9 U/mL (OR 3.672, p=0.042) independently predicted moderate/high disease activity status. Furthermore, tumour M2-PK levels in patients with RA were significantly higher than in those with OA and controls (all p<0.001). However, no differences were found in PKM2 expression in RA and OA synovial tissues as assessed by qPCR, and IHC analysis revealed negligible tumour M2-PK expression in the synovial tissues.

**Conclusion:**

Circulating plasma tumour M2-PK levels may be a clinically useful indicator for evaluating disease activity and RA diagnosis.

## Introduction

Pyruvate kinase (PK) regulates the final step of glycolysis and is responsible for the production of adenosine triphosphate, a crucial source of energy for maintaining cellular activity (1). In mammals, PK is encoded by two genes, PKLR and PKM, and four different isoenzymes (M1, M2, L, and R) are known to exist (2). Among these enzymes, isoenzyme M2 (PKM2) is overexpressed in tumour, embryonic, and actively proliferating cells. PKM2 exists in tetrameric and dimeric forms; the tetrameric form of PKM2 exhibits high affinity for its substrate, phosphoenolpyruvate (PEP), possessing a high activity under physiological PEP concentrations. Therefore, the tetrameric form of PKM2 plays a key role in efficiently converting glucose into pyruvate for glucose utilisation. On the other hand, the dimeric form of PKM2 has a low affinity for PEP, and thus, usually remains inactive under physiological conditions (3).

In tumour cells, intermediate products in the early stages of the glycolytic pathway are metabolised through alternative mechanisms to obtain energy to synthesise nucleic acids, phospholipids, and amino acids, which are important components required for cellular function (4). During this process, PKM2 is converted into a dimeric form as a consequence of post-translational modifications by various tumour proteins; thus, the dimeric form of PKM2 is called tumour M2-PK (5, 6). Previous studies have shown that tumour M2-PK increases in the peripheral circulation of patients with various types of cancers (7). In particular, there is evidence indicating that tumour M2-PK is directly related to tumorigenesis, and its inhibition could have therapeutically beneficial effects against cancers (8). The incorporation of blood tests for tumour M2-PK and conventional tumour markers has been suggested to improve the detection of certain types of cancers (9, 10). Furthermore, the clinical utility of assessing tumour M2-PK level is not only for diagnosis, but it may also serve as a prognostic indicator for determining treatment response, predicting survival, and disease recurrence (11–13). The precise cause of the increase in the dimeric form of PKM2 in tumour cells is still unclear. However, unlike normal cells, it is presumed that this phenomenon could be related to a unique feature of tumour cells, which generate energy by aerobic glycolysis instead of oxidative phosphorylation (14).

Rheumatoid arthritis (RA) is an autoimmune disease characterised by inflammation of the synovial membrane. Uncontrolled chronic inflammation is responsible for the development of irreversible joint deformities (15). In RA, the inflamed synovium contains fibroblast-like synoviocytes (FLSs) and inflammatory cells such as lymphocytes, neutrophils, and macrophages (16). The accumulation and activation of these cells results in the formation of a pannus, which displays a tumour-like phenotype of excessive inflammatory cell accumulation and angiogenesis (17). In fact, a shift toward glycolysis, a characteristic property of cancer cells, has also been identified in FLS from patients with RA and in the synovial tissue of experimental RA (18–20). Therefore, it could be hypothesised that tumour M2-PK, which increases with higher energy requirement, is increased in patients with RA. Based on these shared features, this study was designed to evaluate whether plasma tumour M2-PK level is i) associated with disease activity and blood levels of acute phase reactants such as ESR and CRP, which are the most widely used in clinical care, and ii) higher in patients with RA than in those with osteoarthritis (OA) and controls.

## Materials and methods

### Patients with RA, osteoarthritis, and controls

Plasma samples were collected from 151 patients with RA who had undergone routine laboratory tests in the Department of Rheumatology, Severance Hospital between March 2016 and December 2017. For sample handling, blood collected in EDTA tubes were immediately centrifuged and subsequently stored in a -70°C freezer for enzyme-linked immunosorbent assay (ELISA) experiments. The patients included met the 2010 American College of Rheumatology/European League Against Rheumatism classification criteria for RA (21), and none of the patients had malignancies (either solid or haematologic) or active infections at the time of blood collection. Tumour M2-PK levels in the plasma of 37 patients with OA and 37 controls were compared with those of patients with RA. This study was approved by the Severance Hospital’s Institutional Review Board (4-2017-0761), and the study was conducted keeping with the principles of the 1964 Helsinki Declaration and comparable ethical standards.

### Patient and laboratory data

Patient and laboratory data were acquired on the date of obtaining plasma samples from the patients. Patient demographics included age, sex, seropositivity (rheumatoid factor and/or anti-cyclic citrullinated peptide positivity), the presence of interstitial lung disease (ILD), and disease duration. Disease activity measures included the following disease activity scores: disease activity score in 28 joints (DAS28)-erythrocyte sedimentation rate (DAS28-ESR) and DAS28-C-reactive protein (DAS28-CRP) (22, 23); investigated medications that were used for the treatment of RA were glucocorticoids, conventional synthetic (cs) disease-modifying anti-rheumatic drugs (DMARDs) (methotrexate, leflunomide, sulfasalazine, hydroxychloroquine, and tacrolimus), and biologic DMARDs (bDMARDs) (etanercept, adalimumab, golimumab, infliximab, abatacept, and tocilizumab) (24, 25). Laboratory data included white blood cell, platelet, neutrophil, and lymphocyte counts; erythrocyte sedimentation rate (ESR); and C-reactive protein (CRP), alkaline phosphatase (ALP), aspartate aminotransferase, and alanine aminotransferase levels. Those who were newly diagnosed as RA and was treatment naïve were defined as having new-onset disease, and patients with DAS28-ESR ≥ 3.2 were considered to have moderate/high disease activity, as previously described (26). We defined patients with DAS28-ESR < 3.2 as having remission/low disease activity.

### ELISA for assessment of tumour M2-PK, TNF-α, and IL-6 levels in patients with RA

A commercial ELISA kit (ScheBo Biotech AG, Giessen, Germany) was used to measure plasma M2-PK levels in stored patient samples. In addition, TNF-α (R&D Systems, Minneapolis, MN, USA) and IL-6 (R&D Systems) were evaluated in identical patient samples. The relevant experimental procedures were performed in accordance with the manufacturer’s instructions.

Serial plasma samples of 20 patients with RA who had moderate/high disease activity initially and had remission/low disease activity at follow-up, with a time interval of more than three months, were used to assess changes of tumour M2-PK following the reduction of disease activity.

### Polymerase chain reaction and immunohistochemistry

Total RNA was extracted from 10 knee synovial tissue blocks from patients with RA and OA undergoing total knee replacement surgery using Trizol reagent. Next, a reverse transcription kit (TaKaRa Bio Inc., Kusatsu, Japan) was used for cDNA synthesis, and real-time quantitative PCR (qRT-PCR) analysis was performed using TB Green qPCR Mix (TaKaRa Bio Inc., Kusatsu, Japan) according to the manufacturer’s protocol. We used two different primer sequences of PKM2 for qRT-PCR: (i) forward: 5’-GGAGCGAGATCCCTCCAAAAT-3’, reverse 5’-GGCTGTTGTCATACTTCTCATGG-3’; and ii) forward: 5’-CCACTTGCAATTATTTGAGGAA-3’, reverse 5’-GTGAGCAGACCTGCCAGACT-3). GAPDH was selected as a housekeeping gene (forward: 5’-GGACTGAGGCTCCCACCTTT-3’ and reverse: 5’-CCTGCAGCGTACTCCCCACA-3’). The relative PKM2 mRNA expression was calculated using the 2−ΔΔCT method and normalised to that of GAPDH.

Identical tissue samples were subjected to immunohistochemical (IHC) staining using a commercial antibody against tumour M2-PK (MyBioSource Inc., San Diego, CA, USA). Formalin-fixed, paraffin-embedded tissue sections were de-paraffinised and rehydrated, and immunohistochemistry was performed according to the manufacturer’s instructions.

### Isolation and culture of FLS from synovial fluid

Synovial fluid was obtained from patients with rheumatoid arthritis (n=3) having knee joint swelling by joint aspiration, and was diluted in PBS (1:9), centrifuged at 1500 rpm for 10 minutes. After removing the supernatant, cell pellet was resuspended to complete Dulbecco’s modified Eagle’s medium (DMEM) (10% fetal bovine serum, 100 U/ml penicillin, and 100 mg/ml streptomycin), placed in T25 flask, and incubated in 5% CO_2_ incubator at 37°C. When cell confluency was reached 80~90%, adherent cells were separated, neutralized in complete DMEM, and was spin-downed. Cell pellet was then resuspended for the next passage expansion. The media used for cell culture was changed every 3-4 days (27), and cells in the passage of 3-5 were used for all experiments. Cells used for the experiments were confirmed as FLS by flow cytometry, which was determined as positive for fibroblast marker of CD44 and CD90 and negative for macrophage marker of CD14 (all from BD Biosciences, San Diego, CA, USA) (28–30).

### Stimulation of FLS and cytokine measurement

A total of 1.5×10^5^ cells/well were seeded in a six-well plate, and was cultured with complete DMEM provided with TNF-α, IL-1β (100unit) (Peprotech, Cranbury, NJ, USA), and a PKM2 activator TEPP-46 (10μM) (Cayman Chemical, Ann Arbor, MI, USA) for 72 hours. TEPP‐46 induces the tetramerization of PKM2 and inhibits glycolysis and inflammation (31, 32). Following the end of the culture, the supernatant was collected in 15mL tube and was centrifuged at 1500 rpm for 5 minutes. Cytokines were measured using commercial enzyme-linked immunosorbent assay kits of TNF-α, IL-1β, IFN-γ (Abcam, Cambridge, UK), and IL-6 (Boditech, Chuncheon, South Korea) in the supernatant by duplicate experiments, which was performed according to the manufacturer’s instructions.

### Statistical analysis

Continuous data are shown as medians (interquartile ranges) and categorical variables as numbers (percentages). Pearson’s correlation analysis was conducted for the analysis of the association between tumour M2-PK levels and continuous variables. Statistical differences between continuous variables were assessed using the Mann–Whitney U test or student’s t-test for two groups or by the Kruskal–Wallis test or Analysis of Variance when the groups were more than three, as indicated. Longitudinal changes in plasma tumour M2-PK levels were estimated by a Wilcoxon signed rank test, and derivation of the optimal cut-off of ESR, CRP, and tumour M2-PK was performed using the receiver operating characteristic (ROC) curve analysis. Statistically significant variables in the univariate analysis were subsequently included in the multivariate logistic regression analysis for predicting moderate/high disease activity by applying a forward method. In all statistical analyses, a two-tailed p-value of <0.05 was regarded as significant. Statistical analyses were performed using the MedCalc statistical software version 20.009 (MedCalc Software, Ostend, Belgium).

## Results

### Baseline patient characteristics

The characteristics of the patients are shown in **Table 1**. The median age of the patients was 57.0 of which 118 (78.1%) were women. Patients with seropositive RA accounted for 92.1% of the patients, and ILD was present in 11 patients (7.3%). The median disease duration, DAS28-ESR, and DAS28-CRP level were 61.7 months, 3.2, and 2.3, respectively. For the treatment of patients, 76 (50.3%) patients were undergoing treatment with glucocorticoids, whereas 115 and 56 patients were undergoing treatment with csDMARDs (76.2%) and bDMARDs (37.1%), respectively. The median ESR and CRP values were 36.0 mm/hr and 2.5 mg/L, respectively; the median TNF-α, IL-6, and tumour M2-PK levels were 10.7, 9.1, and 42.9 U/mL, respectively.

**Table 1:** Baseline characteristics of patients with RA.

	Values
Patient demographics	
Age	57.0 (48.0-64.0)
Female sex	118 (78.1)
Seropositive RA	139 (92.1)
Interstitial lung disease	11 (7.3)
Disease duration	61.7 (9.8-145.2)
Disease activity measures	
DAS28-ESR	3.2 (2.4-4.7)
DAS28-CRP	2.3 (1.4-3.8)
Concomitant treatment	
Glucocorticoid	76 (50.3)
DMARDs	121 (80.1)
csDMARDs	115 (76.2)
bDMARDs	56 (37.1)
Laboratory results	
WBC count (/mm^3^)	6250.0 (5165.0-8320.0)
Platelet count (x 1,000/mm^3^)	264.0 (222.3-322.8)
Neutrophil count (/mm^3^)	3660.0 (2875.0-5472.5)
Lymphocyte count (/mm^3^)	1900.0 (1512.5-2255.0)
ESR (mm/h)	36.0 (18.0-67.3)
CRP (mg/L)	2.5 (0.6-11.8)
Alkaline phosphatase (IU/L)	67.0 (56.0-79.8)
AST (IU/L)	19.0 (16.0-24.0)
ALT (IU/L)	15.0 (11.0-23.8)
TNF-α (pg/mL)¶	10.7 (4.5-12.8)
IL-6 (pg/mL)†	9.1 (3.0-27.0)
Tumour M2-PK (U/mL)	42.9 (14.6-104.3)

¶and †Tested in 148 and 146 patients, respectively.

Data are shown as median (interquartile range) or frequency (percentage), as appropriate.

RA, rheumatoid arthritis; DAS28, disease activity score in 28 joints; ESR, erythrocyte sedimentation rate; CRP, C-reactive protein; DMARDs, disease-modifying anti-rheumatic drugs; csDMARDs, conventional synthetic disease-modifying anti-rheumatic drugs; bDMARDs, biologic disease-modifying anti-rheumatic drugs; WBC, white blood cell; AST, aspartate aminotransferase; ALT, alanine aminotransferase; TNF, tumour necrosis factor; IL, interleukin; Tumour M2-PK, dimeric form of pyruvate kinase M2

### Association between tumour M2-PK and disease activity or laboratory data

**Figure 1:**
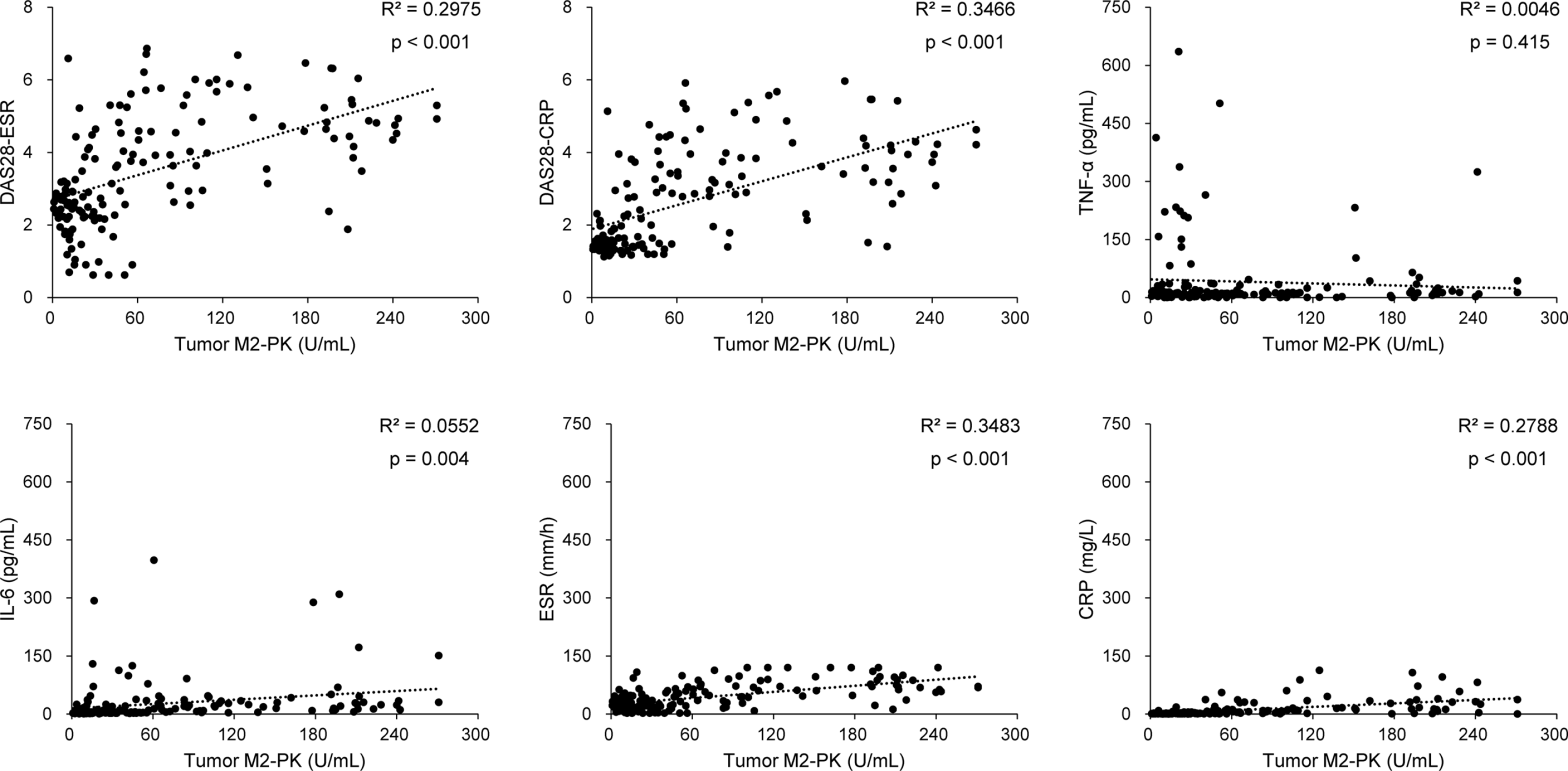
Correlation between tumour M2-PK level and disease activity, TNF-α, IL-6, ESR, and CRP level. Tumour M2-PK levels significantly correlated with disease activity and laboratory results of IL-6, ESR, and CRP, but did not show a correlation with TNF-α. Pearson’s correlation analysis was performed to elucidate the relationship between tumour M2-PK levels and continuous variables. Tumour M2-PK, dimeric form of pyruvate kinase M2; TNF, tumour necrosis factor; IL, interleukin; ESR, erythrocyte sedimentation rate; CRP, C-reactive protein; DAS28: disease activity score in 28 joints.

The tumour M2-PK level was significantly associated with the disease activity measures, DAS28-ESR (r=0.546, 95% confidence interval [CI] 0.423–0.649, p<0.001) and DAS28-CRP (r=0.589, 95% CI 0.474–0.684, p<0.001). In addition, the associations between tumour M2-PK and IL-6, ESR, and CRP were significant; however, there was no association between it and TNF-α (**Figure 1**). Similarly, in patients undergoing DMARD treatment (n=121), a significant relationship was observed between tumour M2-PK level and DAS28-ESR (r=0.539, 95% CI 0.399–0.655, p<0.001) and DAS28-CRP (r=0.590, 95% CI 0.460–0.695, p<0.001) (**Supplementary Table 1**).

**Figure 2:**
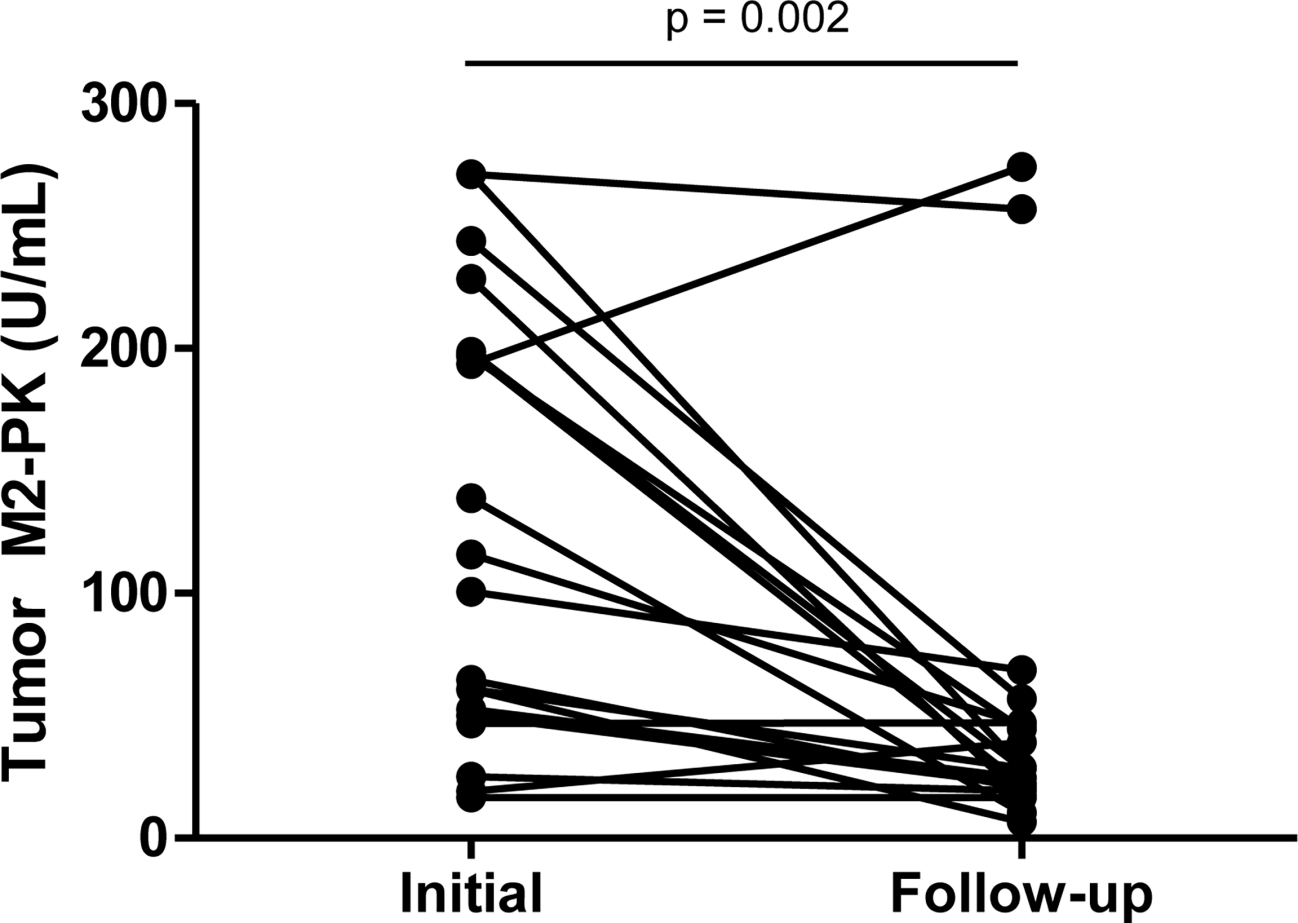
Serial testing of tumour M2-PK level after reduction in disease activity. Tumour M2-PK levels decreased significantly following improvement in disease activity. Changes in plasma tumour M2-PK levels were estimated by the Wilcoxon signed rank test. Tumour M2-PK: dimeric form of pyruvate kinase M2.

Furthermore, repeat testing of tumour M2-PK levels in 20 patients with decreased disease activity revealed a significant decline in tumour M2-PK levels after the reduction in inflammation (p<0.001) (**Figure 2**).

### ROC curve of laboratory variables and logistic regression analysis

**Figure 3:**
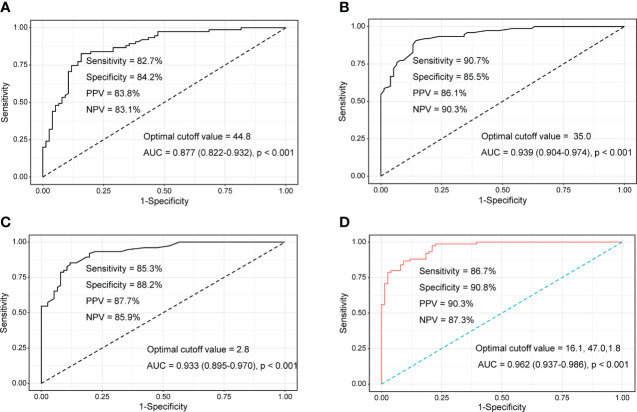
ROC curves of tumour M2-PK, ESR, and CRP to determine moderate/high disease activity and remission/low disease activity. Area under the ROC curve for **(A)** tumour M2-PK, **(B)** ESR, **(C)** CRP, and **(D)** integration of tumour M2-PK, ESR, and CRP for determining optimal cut-off values in discriminating moderate/high RA activity (DAS28-ESR ≥ 3.2) and remission/low disease activity (DAS28-ESR < 3.2). ROC: Receiver operating characteristic; Tumour M2-PK, dimeric form of pyruvate kinase M2; ESR, erythrocyte sedimentation rate; CRP, C-reactive protein; RA, rheumatoid arthritis.

Patients were divided into moderate/high disease activity (n=75) and remission/low disease activity (n=76) groups to evaluate the performance of tumour M2-PK, ESR, and CRP in differentiating disease status. In particular, the median ESR, CRP, and tumour M2-PK levels were highest in those with high disease activity (**Supplementary Table 2**). ROC curve analysis revealed that tumour M2-PK, ESR, and CRP could significantly distinguish between disease status (the AUC was 0.877, 0.939, and 0.933 for tumour M2-PK, ESR, and CRP, respectively). The combination of tumour M2-PK, ESR, and CRP in the ROC curve analysis increased the AUC to 0.962 (**Figure 3**).

In a logistic regression analysis comprised of laboratory results, it was shown that WBC and neutrophil counts; ESR, CRP, ALP, and TNF-α levels; and tumour M2-PK level >43.9 U/mL were significantly associated with moderate/high disease activity. The adjusted analysis revealed that neutrophil count (odds ratio [OR] 1.001, 95% CI 1.000–1.001, p=0.006), ESR (OR 1.108, 95% CI 1.060–1.158, p<0.001), and tumour M2-PK >43.9 U/mL (OR 3.672, 95% CI 1.047–12.878, p=0.042) independently predicted moderate/high disease activity in patients with RA (**Table 2**).

**Table 2:** Univariate and multivariate logistic regression of the association between laboratory results and moderate/high disease activity.

Laboratory results	Univariate analysis			Multivariate analysis		
	OR	95% CI	p value	OR	95% CI	p value
WBC count	1.000	1.000-1.001	<0.001			
Platelet count	1.000	0.999-1.001	0.924			
Neutrophil count	1.001	1.001-1.001	<0.001	1.001	1.000-1.001	0.006
Lymphocyte count	1.000	0.999-1.000	0.044			
ESR	1.108	1.073-1.144	<0.001	1.108	1.060-1.158	<0.001
CRP	1.533	1.307-1.796	<0.001			
Alkaline phosphatase	1.033	1.014-1.053	0.001			
AST	0.958	0.913-1.005	0.079			
ALT	0.992	0.969-1.015	0.487			
TNF-α	1.000	0.996-1.003	0.894			
IL-6	1.018	1.003-1.032	0.016			
Tumour M2-PK > 43.9 U/mL	25.436	10.776-60.040	<0.001	3.672	1.047-12.878	0.042

WBC, white blood cell; ESR, erythrocyte sedimentation rate; CRP, C-reactive protein; AST, aspartate aminotransferase; ALT, alanine aminotransferase; TNF, tumour necrosis factor; IL, interleukin; tumour M2-PK, dimeric form of pyruvate kinase M2; OR, odds ratio; CI, confidence interval.

### Tumour M2-PK levels according to subgroup analysis

**Figure 4:**
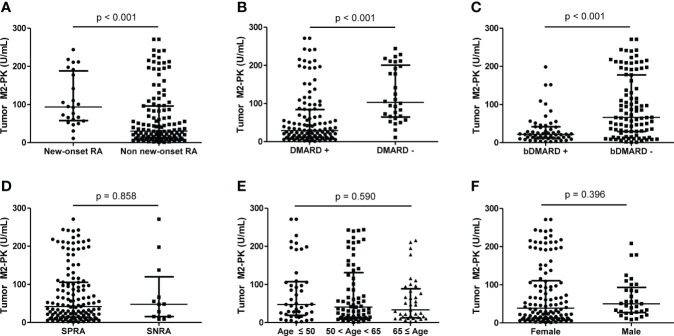
Comparison of tumour M2-PK level based on disease duration, DMARD usage, seropositivity, age, and sex. The differences in tumour M2-PK levels were assessed in RA patients with regard to **(A)** disease duration, **(B)** DMARD usage, **(C)** bDMARD usage, **(D)** serotype, **(E)** age, and **(F)** sex. Mann–Whitney U test and Kruskal–Wallis test was used for the comparison of two groups, and three groups, respectively. The error bars indicate median values and interquartile range. Tumour M2-PK, dimeric form of pyruvate kinase M2; DMARD, disease-modifying anti-rheumatic drug; bDMARD, biologic disease-modifying anti-rheumatic drug; SPRA, seropositive rheumatoid arthritis; SNRA, seronegative rheumatoid arthritis.

To evaluate the difference in tumour M2-PK levels based on disease duration and treatment, patients were classified into those with new-onset (n=24) and those without new-onset disease (n=127). The level of tumour M2-PK was significantly higher in patients with new-onset RA than in those without new-onset disease (p<0.001). Moreover, the tumour M2-PK level was lower in patients who were undergoing any DMARDs treatment or in subjects undergoing bDMARDs treatment (all p<0.001). In contrast, no differences in tumour M2-PK levels were observed with seropositivity, age, and sex (**Figure 4**).

### Tumour M2-PK levels in osteoarthritis and controls

**Figure 5:**
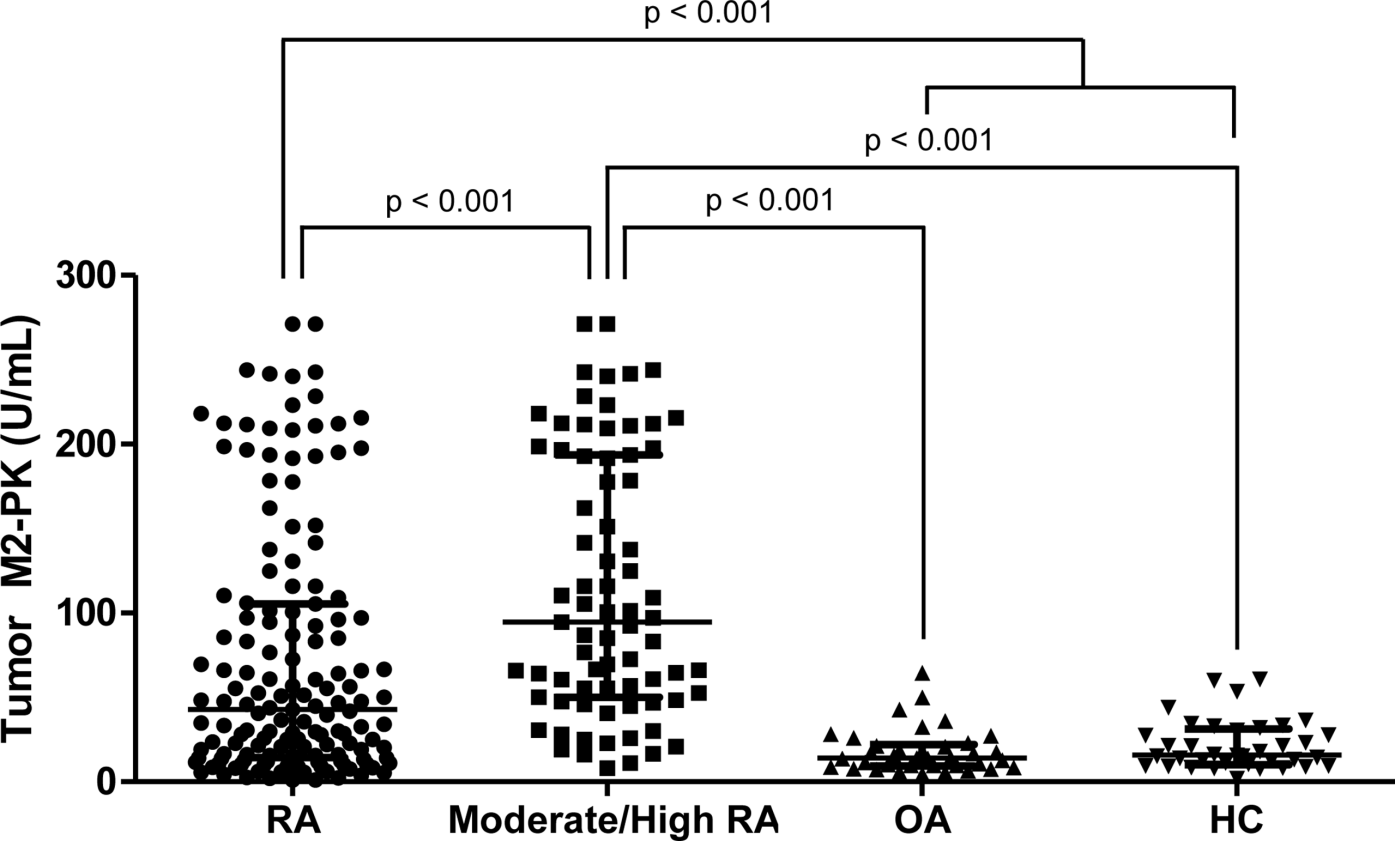
Comparison of tumour M2-PK levels in patients with RA, OA, and controls. Tumour M2-PK levels were compared between patients with RA with moderate/high disease activity, OA, and controls. Differences between the two groups were compared using the Mann–Whitney U test. The error bars indicate median values and interquartile range. Tumour M2-PK, dimeric form of pyruvate kinase M2; RA, rheumatoid arthritis; OA, osteoarthritis; HC, healthy controls.

**Figure 6:**
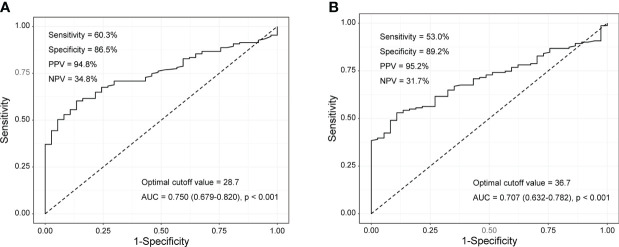
Tumour M2-PK cut-off levels for differentiating RA from OA and controls. Tumour M2-PK levels of 28.7 and 36.7 could be used to differentiate between **(A)** RA and OA patients, and **(B)** RA patients and controls, respectively. The optimal cut-off values were derived using the receiver operating characteristic curve. Tumour M2-PK: dimeric form of pyruvate kinase M2; RA: rheumatoid arthritis; OA: osteoarthritis.

Comparison of tumour M2-PK levels in patients with RA, patients with OA, and controls showed that tumour M2-PK levels were significantly higher in patients with RA than in those with OA or controls (all p<0.001); in particular, the level of tumour M2-PK in patients with RA with moderate/high disease activity was also higher than those in patients with OA and in controls (**Figure 5**). The tumour M2-PK levels were comparable between the OA and control groups. ROC analysis revealed that tumour M2-PK could be used to differentiate patients with RA from those with OA or controls (AUC 0.750, p<0.001 for RA vs. OA and AUC 0.707, p<0.001 for RA vs. controls) (**Figure 6**).

### Expression of PKM2 and tumour M2-PK in synovial tissue

**Figure 7:**
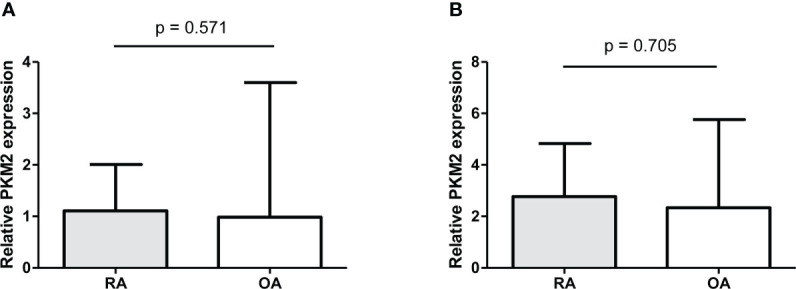
Real-time quantitative polymerase chain reaction of PKM2 in synovial tissues from RA and OA patients. The relative expression of PKM2 in knee synovial tissues was comparable between RA and OA patients (n=10); this was analysed using two different primer sequences: (i) **(A)** and (ii) **(B)**. Relative PKM2 expression was compared by the Mann-Whitney U test. The error bars indicate median values and interquartile range. PKM2: pyruvate kinase isoenzyme M2; RA: rheumatoid arthritis; OA: osteoarthritis.

**Figure 8:**
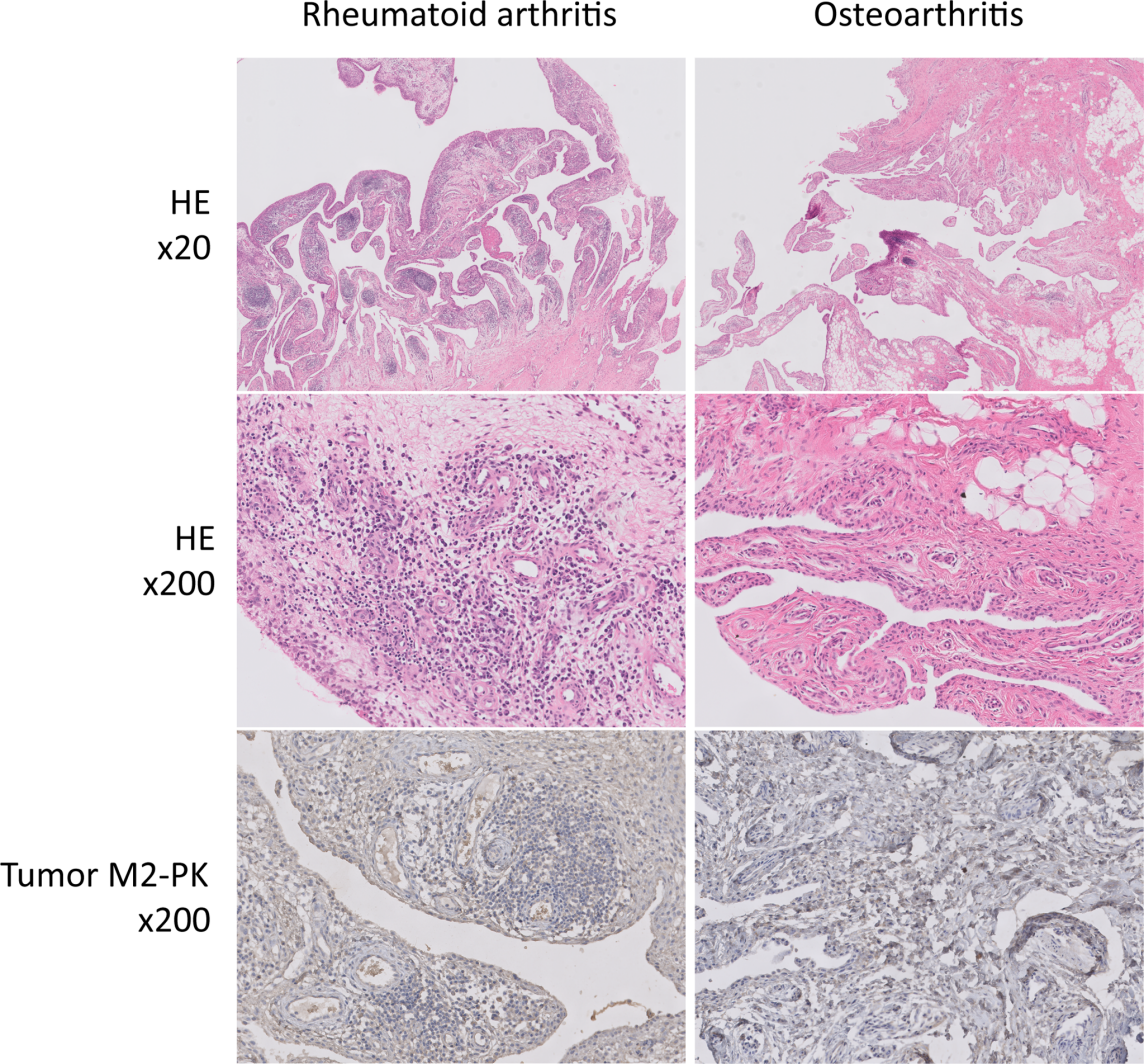
Immunohistochemical analysis of tumour M2-PK in knee synovial tissues of patients with RA and OA. In haematoxylin and eosin-stained synovial tissue from patients with RA, dense infiltration of plasma cells, lymphocytes, and macrophages; germinal centre formation; and villous synovial hyperplasia and hypertrophy were observed. In contrast, the OA synovium showed fatty ingrowth and synovial hyperplasia with myxoid degeneration. Tumour M2-PK immunohistochemical staining in synovial tissues from patients with RA and OA showed no definite expression of local tumour M2-PK. Tumour M2-PK: dimeric form of pyruvate kinase M2; RA, rheumatoid arthritis; OA, osteoarthritis.

The expression of PKM2 was evaluated in the synovial tissues of patients with RA and OA using two different primer sequences; however, there was no difference in the expression of PKM2 in the tissues of patients with RA and OA (**Figure 7A, B**). Meanwhile, IHC staining for tumour M2-PK showed that local expression of tumour M2-PK was not evident in both RA and OA synovium (**Figure 8**).

### Cytokine levels after treatment with TEPP-46

**Figure 9:**
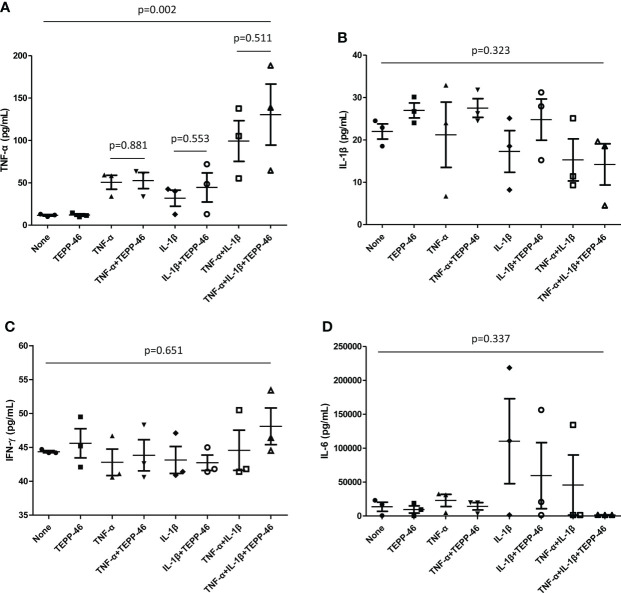
Cytokine assays of supernatant from FLS. FLS (1.5×10^5^ cells) obtained from patients with RA was stimulated by TNF-α or IL-β (100unit) in the presence and absence of TEPP-46 (10μM), a PKM2 activator. After 72 hours, the cytokines of **(A)** TNF-α, **(B)** IL-β, **(C)** IFN-γ, and **(D)** IL-6 in the supernatant was measured by duplicate experiments of enzyme-linked immunosorbent assay. Differences of two groups were evaluated by student’s t-test, whereas Analysis of Variance was used to compared the differences of more than three groups. The error bars indicate mean and SEM. FLS: fibroblast-like synoviocytes; RA, rheumatoid arthritis; TNF, tumour necrosis factor; IL, interleukin; PKM2, pyruvate kinase isoenzyme M2; IFN, interferon.

Cultured FLS were stimulated with TNF-α and IL-1β and the supernatant was analyzed to evaluate whether inflammatory cytokines are increased. When stimulation with both cytokines of TNF-α and IL-1β was performed, the level of TNF-α was significantly higher compared to the absence of these cytokines; however, TNF-α levels did not significantly differ even when TEPP-46 was added to TNF-α, IL-1β, and both TNF-α and IL-1β stimulation (p=0.881, p=0.553, and p=0.511, respectively). In addition, there were no difference in the cytokines of IL-1β, IFN-γ, and IL-6 in the supernatant when TEPP-46 was treated in the presence of TNF-α, IL-1β, and both TNF-α and IL-1β stimulation (**Figure 9**).

## Discussion

In the present study, we investigated whether tumour M2-PK is associated with disease activity in patients with RA and if it could be used to diagnosis RA. First, we found that tumour M2-PK was significantly associated with DAS28-ESR and DAS28-CRP (a composite measure assessing the severity of RA), and ESR and CRP, which decreased after reduction in disease activity. Second, among the laboratory variables included, tumour M2-PK was identified as an independent predictor of moderate/high disease activity in RA. Third, adding tumour M2-PK to ESR and CRP increased the AUC of the ROC curve for differentiating RA with moderate/high from RA with remission/low disease activity to 0.962. Fourth, tumour M2-PK levels were higher in patients with RA than in those with OA or controls, and tumour M2-PK differed between subjects with RA and OA, or controls. Lastly, in contrast to the level of tumour M2-PK in the plasma, the IHC for tumour M2-PK and PCR results for PKM2 in the synovial tissues of patients with RA and OA were comparable. Collectively, these findings suggested that tumour M2-PK in the circulation could be a feasible marker for disease severity and diagnosis in RA.

Alterations in metabolism are now increasingly considered to be closely related to aberrant immune responses, which has drawn special interest in the field of immunometabolism. This is because the upregulation of aerobic glycolysis, which is referred to as the Warburg effect, initially thought to be exclusively found in patients with cancers, has also been demonstrated in patients with autoimmune diseases (33). In particular, accumulating evidence emphasises that the Warburg effect is present in the affected joints in RA and may be a potential treatment target in modulating articular inflammation (20, 34, 35). In this context, it is possible that plasma tumour M2-PK is associated with disease activity in RA through either glycolysis- or non-glycolysis-dependent mechanisms (36). First, tumour M2-PK may be a marker of increased glycolysis in RA. In general, highly proliferating cells, which include FLS and immune cells in RA, are thought to have an increased energy requirement (18). Therefore, facilitation of energy production *via* increased glycolysis is required in the RA microenvironment to meet the increased energy demand. Second, PKM2 is also involved in the activation of key transcription factors in RA, such as signal transducer and activator of transcription (STAT) 1 and 3, hypoxia-inducible factor-1α, and nuclear factor-κB, which are closely linked to the inflammatory signalling pathway in RA (36). Notably, nuclear PKM2 exists in a dimeric form and acts as a protein kinase that influences the transcription of inflammation-related genes, indicating that it may represent heightened inflammation in RA (37). Third, tumor M2-PK was shown to promote angiogenesis in an *in vitro* analysis, which is also thought to be a characteristic feature observed within the inflamed RA joints (38, 39).

Based on the reasons provided above, we postulated that the tumour M2-PK level could parallel the activity of RA and would be lower in patients with OA and controls than in patients with RA. Supporting our hypothesis, plasma tumour M2-PK levels exhibited a significant correlation with disease activity and were predictive of disease activity status in RA, which was also found to be higher in RA patients than in those with OA and controls. Importantly, a longitudinal assessment of tumour M2-PK demonstrated that its decrease reflected a reduction in disease activity. ESR and CRP were the most commonly performed laboratory tests to estimate disease improvement or aggravation, and combining tumour M2-PK with these tests in the ROC curve analysis for the discrimination of disease severity showed an excellent performance, with an AUC which was higher than that of ESR and CRP alone; this implies that it could have an additional clinical value in measuring RA activity. However, it is well known that tumour M2-PK increase is relevant for the extent of disease and prognosis of cancers, especially in the gastrointestinal tract (40, 41). Thus, patients with cancer were excluded from our study. However, in patients with RA who show increased tumour M2-PK, it is apparent that the possibility of malignancy should be examined thoroughly before clinical application because malignancies could affect tumour M2-PK levels.

Similar to the results of this study, a recent publication by Han et al. demonstrated that extracellular PKM2 is increased in patients with RA and is related to disease activity. It was also demonstrated that extracellular PKM2 level was associated with radiographic progression in a subset of patients with early RA and recombinant PKM2 was involved in osteoclastogenesis in an *in vitro* analysis (42). Nonetheless, our study is unique that the clinical utility of tumour M2-PK in assessing the disease activity of RA has been analyzed detailedly in a separate cohort using ROC and logistic analyses. In addition, comparison of tumour M2-PK level in RA, OA, and controls and serial assessment of its level confirms that measuring plasma tumor M2-PK may be a practical test in discriminating RA and OA, as well as in the management of patients with RA. Of note, in our *in vitro* analysis using FLS, we did not observe a significant reduction of inflammatory cytokines when PKM2 activator TEPP-46 was used. This finding seems to be partly explained by a previous observation that secretion of tumour M2-PK are more likely to be affected by monocytes than FLS (42), implying immune cells such as T-cells and monocyte/macrophages might be a greater source of elevated tumour M2-PK in patients with RA (43). On the other hand, a complex association of inflammation and metabolic deregulation could be also considered as a reason for this insignificant results. Interestingly, a previous publication revealed that the decrease of inflammation could intrinsically modify immunometabolism in RA (44). Overall, it is apparent that the dynamics of dimeric and tetrameric form of PKM2 and the role of tumour M2-PK in potentiating inflammation in RA deserves further investigation.

An earlier study showed that immunohistochemistry of PKM2 is enhanced in cells residing in the lining and sub lining of synovial tissues of patients with RA compared to those of patients with OA (45). However, in this study, qPCR analysis of PKM2 in synovial tissues showed that there was no significant difference in PKM2 expression between RA and OA. These discrepant results could be attributed to the fact that PKM2 IHC staining appears to be limited to a specific region within the synovium and may not sufficiently reflect the degree of local inflammation in the synovium. Alternatively, the increase in inflammatory mediators which was also observed in the OA microenvironment and changes in RA synovial tissue pathologic findings even after active treatment, could also contribute to the comparable PKM2 qPCR results (46, 47). IHC staining of tumour M2-PK in RA and OA synovium revealed that its expression was not noticeable. The relatively low proportion of dimeric PKM2 relative to its tetrameric isoform, even when there is a high energy demand, as shown in cancer cells, might explain the negligible tumour M2-PK staining in RA and OA synovium (48). Finally, it is possible that PKM2 and tumour M2-PK expression were affected by formalin fixation. Accordingly, in-depth research is necessary to further elucidate the role of tumour M2-PK and PKM2 in tissues from patients with RA.

Our study has several limitations. First, both incident and prevalent cases of RA were included, and the proportion of patients with new-onset RA was relatively low. Second, a detailed assessment of the impact of the treatment regimen on plasma tumour M2-PK levels could not be performed because of the present study design. Third, only the level of tumour M2-PK was analysed, and its expression relative to that of PKM2 was not evaluated. Fourth, although patients with RA with moderate//high disease activity had significantly higher levels of tumour M2-PK level than those with OA and controls, the number of patients were small and could be considered exploratory. Finally, the underlying pathophysiology of increased plasma tumour M2-PK in RA and its direct pathogenic role could not be determined. Future studies are required to better understand whether tests evaluating changes in glucose metabolism can be used as surrogate markers of RA severity.

### Conclusions

In conclusion, we found that plasma tumour M2-PK levels significantly correlated with disease activity and independently predicted disease severity in RA, which decreased with reduction in disease activity. Furthermore, plasma tumour M2-PK was higher in patients with RA than in patients with OA and controls. Our results suggest that the assessment of plasma tumour M2-PK levels might be a clinically useful indicator for evaluating disease activity and RA diagnosis.

## Data availability statement

The raw data supporting the conclusions of this article will be made available by the authors, without undue reservation.

## Ethics statement

The studies involving human participants were reviewed and approved by Severance Hospital’s Institutional Review Board. Written informed consent for participation was not required for this study in accordance with the national legislation and the institutional requirements.

## Author contributions

Conceptualization, SSA and HK. Methodology, SSA, HK and YP. Software, SSA and HK. Validation, SSA. Formal analysis, SSA. Investigation, SSA, HK and YP. Resources, SSA, HK and YP. Data curation, SSA. Writing—original draft preparation, SSA, HK and YP. Writing—review and editing, SSA, HK and YP. Visualization, SSA and HK. Supervision, YP. Project administration, SSA, HK and YP. Funding Acquisition, SSA. All authors have read and agreed to the final version of the manuscript. All authors contributed to the article and approved the submitted version.

## Funding

This study was supported by a new faculty research seed money grant of Yonsei University College of Medicine for 2022 (2022-32-0056).

## Conflict of interest

The authors declare that the research was conducted in the absence of any commercial or financial relationships that could be construed as a potential conflict of interest.

## Publisher’s note

All claims expressed in this article are solely those of the authors and do not necessarily represent those of their affiliated organizations, or those of the publisher, the editors and the reviewers. Any product that may be evaluated in this article, or claim that may be made by its manufacturer, is not guaranteed or endorsed by the publisher.
