# Supplementary material for: Assessment of disease activity in patients with rheumatoid arthritis using plasma tumour M2-pyruvate kinase test
Source: Front Immunol. 2022 Aug 18;13:901555. doi: 10.3389/fimmu.2022.901555 (PMC9433835; doi:10.3389/fimmu.2022.901555)
Supplement: Supplementary file 1 [file Table_1.docx]

Supplementary Material

# Supplementary Table 1. Relationship between tumour M2-PK levels and disease activity,

# TNF-α, IL-6, ESR, and CRP levels in patients undergoing DMARD treatment (n=121)

|  | Correlation  coefficient (r) | 95% confidence interval | p-value |
| --- | --- | --- | --- |
| DAS28-ESR | 0.539 | 0.399-0.655 | <0.001 |
| DAS28-CRP | 0.590 | 0.460-0.695 | <0.001 |
| TNF-α | -0.023 | -0.201-0.157 | 0.806 |
| IL-6 | 0.182 | 0.001-0.351 | 0.049 |
| ESR | 0.583 | 0.452-0.690 | <0.001 |
| CRP | 0.641 | 0.522-0.735 | <0.001 |

Tumour M2-PK, dimeric form of pyruvate kinase M2; TNF, tumour necrosis factor; IL, interleukin;

ESR, erythrocyte sedimentation rate; CRP, C-reactive protein; DMARD, disease-modifying anti-

rheumatic drug; DAS28: disease activity score in 28 joints
