# Supplementary material for: Assessment of disease activity in patients with rheumatoid arthritis using plasma tumour M2-pyruvate kinase test
Source: Front Immunol. 2022 Aug 18;13:901555. doi: 10.3389/fimmu.2022.901555 (PMC9433835; doi:10.3389/fimmu.2022.901555)
Supplement: Supplementary file 2 [file Table_2.docx]

Supplementary Material

**Supplementary Table 2. Levels of ESR, CRP, and tumour M2-PK according to disease activity**

**status**

|  | Remission (n=51) | Low disease activity (n=25) | Moderate disease activity (n=47) | High disease activity (n=28) | p-value |
| --- | --- | --- | --- | --- | --- |
| ESR (mm/hr) | 17.0 (5.3-20.8) | 34.0 (26.3-45.3) | 56.0 (41.5-70.8) | 81.5 (69.5-99.5) | <0.001 |
| CRP (mg/L) | 0.5 (0.3-1.1) | 1.0 (0.6-4.4) | 9.1 (4.6-16.4) | 29.1 (6.2-41.8) | <0.001 |
| Tumour M2-PK (U/mL) | 19.0 (10.9-33.1) | 14.1 (7.9-43.4) | 85.0 (46.1-197.3) | 105.6 (65.2-185.1) | <0.001 |

Data are presented as median (interquartile range).

ESR, erythrocyte sedimentation rate; CRP, C-reactive protein; Tumour M2-PK, dimeric form of

pyruvate kinase M2
